# Association Rule Mining and Prognostic Stratification of 2-Year Longevity in Octogenarians Undergoing Endovascular Therapy for Lower Extremity Arterial Disease: Observational Cohort Study

**DOI:** 10.2196/17487

**Published:** 2020-12-01

**Authors:** Jing-Yi Jhang, I-Shiang Tzeng, Hsin-Hua Chou, Shih-Jung Jang, Chien-An Hsieh, Yu-Lin Ko, Hsuan-Li Huang

**Affiliations:** 1 Division of Cardiology Department of Internal Medicine Taipei Tzu-Chi Hospital, Buddhist Tzu Chi Medical Foundation New Taipei Taiwan; 2 Department of Research Taipei Tzu Chi Hospital Buddhist Tzu Chi Medical Foundation New Taipei Taiwan; 3 School of Medicine Tzu Chi University Hualien Taiwan; 4 School of Post-Baccalaureate Chinese Medicine Tzu Chi University Hualien Taiwan

**Keywords:** endovascular therapy, lower extremity arterial disease, octogenarians, longevity, association rules, older people, prognosis, risk, medical informatics, clinical informatics

## Abstract

**Background:**

Two-year longevity is a crucial consideration in revascularization strategies for patients with symptomatic lower extremity arterial disease (LEAD). However, factors associated with 2-year longevity and risk stratification in octogenarians or nonagenarians have been underreported.

**Objective:**

This paper aims to investigate the associated variables and stratify the 2-year prognosis in older patients with LEAD.

**Methods:**

We performed logistic regression and association rule mining based on the Apriori algorithm to discover independent variables and validate their associations with 2-year longevity. Malnutrition, inflammation, and stroke factors were identified. C statistics and Kaplan-Meier analysis were used to assess the impact of different numbers of malnutrition, inflammation, and stroke factors on 2-year longevity.

**Results:**

We recruited a total of 232 octogenarians or nonagenarians (mean age 85 years, SD 4.2 years) treated with endovascular therapy. During the study period, 81 patients died, and 27 of those (33%) died from a cardiac origin within 2 years. Association rules analysis showed the interrelationships between 2-year longevity and the neutrophil-lymphocyte ratio (NLR) and nutritional status as determined by the Controlling Nutritional Status (CONUT) score or Geriatric Nutritional Risk Index (GNRI). The cut-off values of NLR, GNRI, and CONUT were ≥3.89, ≤90.3, and >3, respectively. The C statistics for the predictive power for 2-year longevity were similar between the CONUT score and the GNRI-based models (0.773 vs 0.760; *P*=.57). The Kaplan-Meier analysis showed that 2-year longevity was worse as the number of malnutrition, inflammation, and stroke factors increased from 0 to 3 in both the GNRI-based model (92% vs 68% vs 46% vs 12%, respectively; *P*<.001) and the CONUT score model (87% vs 75% vs 49% vs 10%, respectively; *P*<.001). The hazard ratio between those with 3 factors and those without was 18.2 (95% CI 7.0-47.2; *P*<.001) in the GNRI and 13.6 (95% CI 5.9-31.5; *P*<.001) in the CONUT score model.

**Conclusions:**

This study demonstrated the association and crucial role of malnutrition, inflammation, and stroke factors in assessing 2-year longevity in older patients with LEAD. Using this simple risk score might assist clinicians in selecting the appropriate treatment.

## Introduction

With the aging of the population and improvements in the quality of medical care, physicians encounter an ever-increasing number of older patients with advanced forms of lower extremity arterial disease (LEAD) [1,2]. The results of the Bypass Versus Angioplasty in Severe Ischaemia of the Leg (BASIL) study [3] and the American Heart Association and American College of Cardiology guidelines [4] suggested bypass surgery as an appropriate first-line revascularization procedure for chronic limb-threatening ischemia (CLTI) in patients with a life expectancy of more than 2 years. However, advanced age is associated with increased perioperative and postoperative mortality after vascular operations because of the presence of multiple comorbidities [5-7]. Some studies have reported that endovascular therapy (EVT) in older patients is safe and effective for lifestyle-limiting intermittent claudication and could be an effective alternative treatment for CLTI [1,6,8,9].

Although 2-year longevity is a crucial consideration in revascularization strategies in patients with symptomatic LEAD, this measure should be used with caution in older people with an inherently shorter life expectancy, and the factors associated with 2-year longevity and the prognostic stratification in these patients are unclear.

Apriori algorithm–based association rules analysis (ARA) is widely used to analyze interesting statistical correlations hidden in sets of multidimensional data and might facilitate the process of disease management [10-12].

The aim of this study was to investigate the factors predicting 2-year life expectancy in octogenarians or nonagenarians with LEAD in order to help clinicians care for these patients.

## Methods

### Study Design

This was a single-center observational cohort study and was approved by the institutional review board (approval No. 06-X18-098) of Taipei Tzu-Chi Hospital, New Taipei City, Taiwan. We obtained informed consent from all participants.

### Study Population

Data were extracted from the Tzuchi Registry of Endovascular Intervention for Peripheral Artery Disease, which is a single-center observational registry of patients who have undergone EVT for LEAD starting from July 2005. This retrospective investigation included patients older than 80 years with symptomatic LEAD who were treated between July 2005 and June 2017.

**Figure 1:**
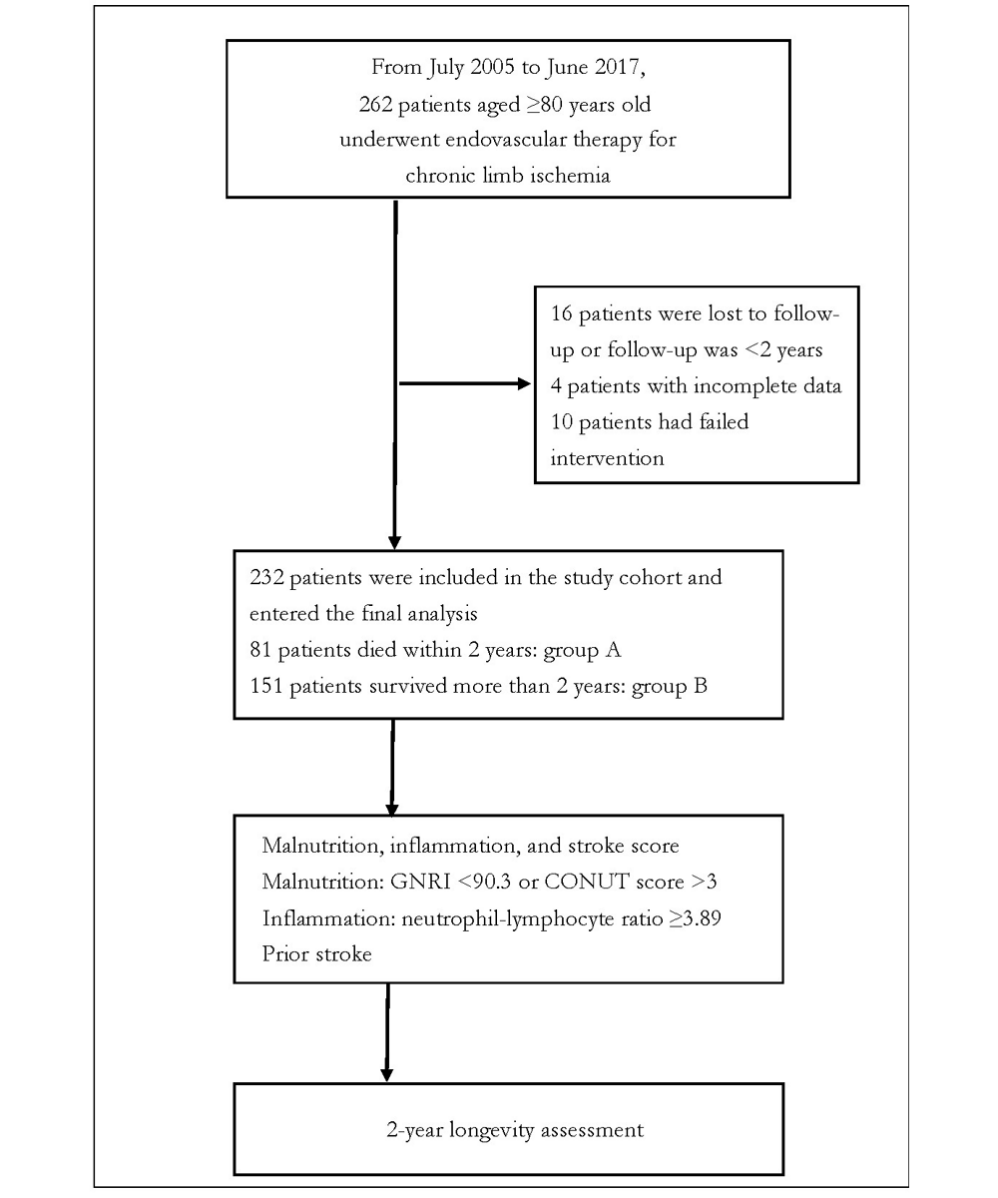
Flowchart of the inclusion of the study participants. CONUT: Controlling Nutritional Status; GNRI: Geriatric Nutritional Risk Index.

Patients were considered eligible for enrollment if they were candidates for EVT for atherosclerotic LEAD and had given their consent to participate in the study. Excluded from the study were patients with nonatherosclerotic LEAD, acute limb ischemia, overwhelming foot infection, and a follow-up duration of less than 2 years. Figure 1 depicts patients’ enrollment in this study. All study procedures were in line with the principles outlined in the Helsinki Declaration.

The detailed definitions of demographic variables and the pre-EVT assessments, including clinical examination, hemodynamic evaluation, and anatomical assessment of the target limb, have been described previously [8,13].

### Interventions

Endovascular therapy was performed by experienced interventional cardiologists according to the Trans-Atlantic Inter-Society Consensus II guideline recommendations [14]. The detailed procedures of EVT have also been described previously [8,13]. Most of the study participants underwent plain balloon angioplasty alone or bare-metal nitinol stent implantation. With the advancement in technology and the invention of new devices, atherectomy and drug-eluting devices have been introduced to optimize the angiographic results and improve vessel patency. Antiplatelet therapy, anticoagulant regimens, and other medications after EVT depended on the physician’s discretion and the patient’s condition.

### Biochemical Markers

Blood samples were obtained at admission or before EVT for the measurement of serum albumin, fasting blood sugar, glycohemoglobin, C-reactive protein (CRP), complete blood count (CBC), and differential counts for the determination of immune-inflammatory variables; this included the neutrophil-lymphocyte ratio (NLR), the platelet-lymphocyte ratio (PLR), and the systemic immune-inflammation index (SII), defined as (platelet count × neutrophil count)/lymphocyte count. The total cholesterol, triglyceride, and high- and low-density lipoprotein cholesterol levels were measured at admission, and the most recent values obtained within 2 months before the procedure were also examined.

### Nutritional Status

The Geriatric Nutritional Risk Index (GNRI) and the Controlling Nutritional Status (CONUT) score are widely used in various clinical settings in the field of malnutrition [15,16]. They have been reported as prognostic predictors in patients with LEAD [17]. The CONUT score includes serum albumin, total cholesterol levels, and total lymphocyte count. CONUT scores of 0 to 1 indicate a normal nutritional status, scores of 2 to 4 indicate mild risk, scores of 5 to 8 indicate moderate risk, and scores of 9 to 12 indicate severe risk of malnutrition. GNRI was also calculated using the following equation: GNRI = (14.89 serum albumin [g/dL]) + (41.7 × [body weight/ideal body weight]) [18].

### Association Rule Analysis and Grouped Matrix Method

ARA was initially designed to detect and extract useful information from large-scale business databases [10,11]. Recently, this method has been widely applied in clinical medicine to facilitate disease diagnosis and prevention and improve the survival rate [19,20]. The fundamental concept of ARA usually takes the form of A → B, which means A implies B for any set of variables A and B. Briefly, the implication of ARA is co-occurrence and not causality. For clinical applications, the frequent association between combinations of variables is used to determine the expected prevalence (or occurrence) of disease over random chance. Three core values are used to determine interesting or useful rules: support, confidence, and lift.

Support is defined as P(A,B) (ie, the joint probability of A and B), that is, the prevalence of both A and B co-occurring. Confidence is the conditional probability that B occurs, given that A is already present. The lift (presented as the observed to expected ratio) is expressed as P(A,B) to P(A) P(B), which is a measure of the level of dependence between A and B. A lift of 1 indicates that A and B are independent without any association with each other. In ARA, the sets of A and B are restricted to a single variable because many possible combinations of associations exist between variables (122 in this study). The grouped matrix method creates nested groups of rules via clustering. The nested groups form a hierarchy, which can interactively explore multiple variables of rules that precede the following variable.

### Definitions and Outcome End Points

We defined procedural success as the successful restoration of blood flow, with evidence of at least one preexisting or reestablished crural vessel to the foot. The lesion score is the sum of the scores of the diseased lower-extremity blood vessels. A score of 1 point each was given for the iliac, femoropopliteal, anterior tibial, posterior tibial, and peroneal arteries [21].

Nonambulatory patients were defined as patients who used wheelchairs or patients with a bedridden status. Cardiovascular death included sudden cardiac arrest or death caused by myocardial infarction, stroke, lethal arrhythmia, decompensated heart failure, valvular heart disease, and aortic or other vascular diseases.

The outcome end point was 2-year survival with risk stratification according to the determining factors of 2-year longevity.

### Follow-up

After EVT, we performed scheduled follow-ups at 1 month and every 3 months with clinical assessment and duplex ultrasound evaluations. Repeat EVT was performed if symptoms recurred. Major events (death, amputation, and all vascular events) were documented at the follow-up visits. If data of office follow-up visits were not available, alternate data sources included telephone interviews, medical records, and the referring physician. The follow-up closing date was December 31, 2018.

### Statistical Analysis

Statistical analysis was performed using SPSS Statistics (version 22.0; IBM Corp) and MedCalc statistical software (version 18.11.3; MedCalc Software). Descriptive statistics were presented as frequency (percent) for categorical variables and mean (standard deviation) for continuous variables. Discrete and categorical data were analyzed using the Pearson chi-square test. The parametric continuous variables were statistically analyzed and compared between groups using an independent *t* test, whereas the Mann-Whitney U test was used to analyze nonparametric continuous and ordinal data. CBC, NLR, PLR, SII, and CRP levels were presented as medians and interquartile ranges and were logarithmically transformed before statistical analysis. A receiver operating characteristic (ROC) curve was used to determine the cut-off values of the NLR, GNRI, and CONUT score for 2-year longevity prediction. The area under the curve (AUC) of these variables was compared using the DeLong method. Cox proportional hazard model analysis was performed to identify the independent predictors associated with 2-year longevity. The predictive performance levels of the combination model for 2-year longevity in C statistics were also compared using the DeLong method. A Kaplan-Meier analysis was conducted to compare 2-year longevity using the number of malnutrition, inflammation, and stroke factors. *P* values of <.05 were considered statistically significant.

We validated the associations of malnutrition and inflammation with 2-year longevity using ARA [10,11,22] and by visualizing associations using R software (version 3.4.3; The R Foundation for Statistical Computing). The procedure can be conveniently fitted using the R package “arules.” The visualizing association rules can be directly applied using the R package “arulesViz.” A total of 15 potential variables were investigated for association rules, as determined by the minimum requirements of a support degree of ≥20% and confidence of ≥80% in this study.

## Results

We treated 262 older patients with EVT from July 2005 to June 2017. Of these, 30 were not eligible for analysis, 16 were lost to follow-up or had a follow-up duration of less than 2 years, 4 had incomplete data, and 10 had failed EVT. The remaining 232 patients were divided into 2 groups based on whether they had a survival duration of ≥2 years. Group A included 81 patients who died (27/81, 33% died from cardiac origin) within 2 years after the index EVT, while group B was made up of 151 patients who survived for more than 2 years.

Table 1 summarizes the baseline demographics of the 2 groups. Patients in group A had a significantly higher incidence of congestive heart failure (CHF), cerebrovascular accident (CVA), dialysis, CLTI, and nonambulatory status than those in group B. BMI, serum albumin level, cholesterol level, and GNRI were significantly higher in group B patients. Higher levels of inflammatory markers (white blood cell [WBC] count, NLR, PLR, SII, and CRP level) and CONUT scores were more frequently observed in group A than in group B.

Table 2 shows the lesion characteristics of the treated limbs in the 2 groups. Group A patients had a lower ankle-brachial pressure index and a higher incidence of CLTI than group B patients. There were no differences between the 2 groups in lesion distribution, complexity, isolated or multilevel EVTs, and stenting rate.

**Table 1:** Patient demographics.

Factors	All patients	Group A (2-year death)	Group B (2-year survival)	*P* value
Patients, N	232	81	151	N/A^a^
Age (years), mean (SD)	85.4 (4.2)	85.8 (4.0)	85.3 (4.3)	.41
Sex (male), n (%)	109 (47)	36 (44)	73 (48)	.57
Diabetes mellitus, n (%)	130 (56)	51 (62)	79 (52)	.12
Hypertension, n (%)	201 (87)	72 (89)	129 (85)	.46
Coronary artery disease, n (%)	93 (40)	32 (40)	61 (40)	.90
Congestive heart failure, n (%)	39 (17)	19 (24)	20 (13)	.047
Cerebrovascular accident, n (%)	52 (22)	29 (36)	22 (15)	<.001
Dialysis dependence, n (%)	57 (25)	27 (33)	30 (20)	.02
Smoking history, n (%)	58 (25)	19 (24)	39 (26)	.69
Atrial fibrillation, n (%)	49 (21)	20 (25)	29 (19)	.33
Hyperlipidemia, n (%)	95 (41)	31 (39)	64 (42)	.59
Ambulatory status, n (%)	83 (36)	16 (20)	67 (44)	<.001
CLTI^b^, n (%)	192 (83)	78 (96)	114 (76)	<.001
Body mass index (kg/m^2^), mean (SD)	23.1 (3.5)	22.3 (3.5)	23.6 (3.4)	.006
Cholesterol (mg/dL), mean (SD)	159 (39)	150 (39)	163 (39)	.01
Triglyceride (mg/dL), mean (SD)	117 (70)	111 (63)	119 (73)	.46
Glycohemoglobin (%), mean (SD)	6.51 (1.50)	6.70 (1.68)	6.42 (1.41)	.21
Hematocrit (%), median (IQR)	33.7 (29.8-37.2)	33.5 (29.9-36.5)	33.9 (29.8-38.1)	.10
White blood cell count (10^9^/L), median (IQR)	7.230 (5.673-9.080)	7.970 (6.720-9.965)	6.850 (5.120-8.200)	.001
Platelet count (10^3^/μL), median (IQR)	208 (164-255)	217 (169-278)	205 (156-245)	.07
Neutrophil-lymphocyte ratio, median (IQR)	3.62 (2.34-5.62)	5.03 (3.31-7.02)	3.03 (2.04-4.64)	<.001
Platelet-lymphocyte ratio, median (IQR)	145 (114-219)	173 (120-243)	135 (107-195)	.002
Systemic immune-inflammation index^c^, median (IQR)	716 (461-1272)	1083 (568-1835)	598 (383-972)	<.001
C-reactive protein (mg/dL), median (IQR)	1.25 (0.31-4.40)	3.08 (0.77-8.20)	0.90 (0.19-2.92)	<.001
Albumin (g/dL), mean (SD)	3.10 (0.65)	2.78 (0.63)	3.26 (0.59)	<.001
CONUT^d^ score, mean (SD)	4.74 (2.97)	6.26 (2.81)	3.93 (2.72)	<.001
Geriatric Nutritional Risk Index, mean (SD)	89.3 (12.5)	83.1 (12.3)	92.6 (11.3)	<.001
Follow-up time (days), median (IQR)	971 (389-1575)	229 (79-438)	1360 (1008-1942)	<.001

^a^N/A: not applicable.

^b^CLTI: chronic limb-threatening ischemia.

^c^Systemic immune-inflammation index was defined as (platelet count × neutrophil count)/lymphocyte count.

^d^CONUT: Controlling Nutritional Status.

**Table 2:** Lesion and interventional procedure characteristics.

Factors	All limbs	Group A (2-year death)	Group B (2-year survival)	*P* value
Limbs, n	232	81	151	N/A^a^
Claudication, n (%)	40 (17)	3 (4)	37 (25)	.001
Resting pain, n (%)	51 (22)	19 (23)	32 (21)	N/A
Unhealing ulcer, n (%)	109 (47)	44 (54)	65 (43)	N/A
Gangrene, n (%)	32 (14)	15 (19)	17 (11)	N/A
ABI^b^ of affected limbs, mean (SD)	0.54 (0.33)	0.51 (0.37)	0.55 (0.31)	.40
ABI of affected limbs excluding ≥1.4, mean (SD)	0.45 (0.19)	0.41 (0.18)	0.47 (0.19)	.02
Iliac lesions, n (%)	30 (13)	9 (11)	21 (14)	.98
Femoropopliteal lesions , n (%)	167 (72)	61 (75)	106 (70)	.35
Below-the-knee lesions, n (%)	170 (73)	61 (75)	109 (73)	.63
Poor runoff, n (%)	192 (84)	67 (85)	125 (83)	.77
Stenting, n (%)	112 (48)	37 (46)	75 (50)	.53
Lesion score, mean (SD)	3.18 (0.98)	3.30 (0.97)	3.11 (0.98)	.16

^a^N/A: not applicable.

^b^ABI: ankle-brachial index.

Compared with WBCs, SII, and PLR, NLR had the largest AUC (0.648, 0.692, 0.621, and 0.703, respectively) (Multimedia Appendix 1) and was the only significant inflammatory variable for 2-year longevity estimation after multivariate analysis. The cut-off value of NLR for 2-year longevity was 3.89. The AUCs for 2-year longevity estimation by the CONUT score or GNRI were statistically significant (*P*<.001); however, there was no difference between the 2 measures (0.725 vs 0.722; *P*=.90). The cut-off values for the GNRI and the CONUT score for 2-year longevity were 90.3 and >3, respectively. Detailed patient demographics and lesion characteristics are provided in Multimedia Appendices 2 and 3.

Table 3 shows the results of 2 separate multivariate analyses examining the predictors of 2-year longevity (model 1 was adjusted for ambulatory status, CHF, CVA, CLTI, dialysis, NLR, and total cholesterol; model 2 was adjusted for the covariates, except that BMI was used to replace total cholesterol). CVA, NLR, GNRI, and the CONUT score were independent factors of 2-year longevity.

**Table 3:** Results of multivariate logistic regression analysis.

Variables	Model 1 (GNRI^a^ + NLR^b^)		Model 2 (CONUT^c^ + NLR)	
	HR^d^ (95% CI)	*P* value	HR (95% CI)	*P* value
Ambulatory status	0.718 (0.291-1.773)	.47	0.776 (0.320-1.884)	.58
Congestive heart failure	2.167 (0.950-4.945)	.07	1.932 (0.827-4.513)	.13
Cerebrovascular accident	2.763 (1.231-6.202)	.01	2.577 (1.169-5.678)	.02
C-reactive protein	1.135 (0.890-1.149)	.31	1.193 (0.933-1.527)	.16
CLTI^e^	1.961 (0.425-9.043)	.39	2.439 (0.535-11.110)	.25
Dialysis	1.774 (0.816-3.855)	.15	1.711 (0.807-3.626)	.16
Cholesterol	1.007 (0.998-1.016)	.16	N/A^f^	N/A
Body mass index	N/A	N/A	1.087 (0.979-1.206)	.12
CONUT score >3	N/A	N/A	2.718 (1.217-6.073)	.02
NLR >3.89	2.679 (1.312-5.470)	.007	2.532 (1.236-5.187)	.01
GNRI <90.3	3.071 (1.447-6.516)	.003	N/A	N/A

^a^GNRI: Geriatric Nutritional Risk Index.

^b^NLR: neutrophil-lymphocyte ratio.

^c^CONUT: Controlling Nutritional Status.

^d^HR: hazard ratio.

^e^CLTI: chronic limb-threatening ischemia.

^f^N/A: not applicable.

**Figure 2:**
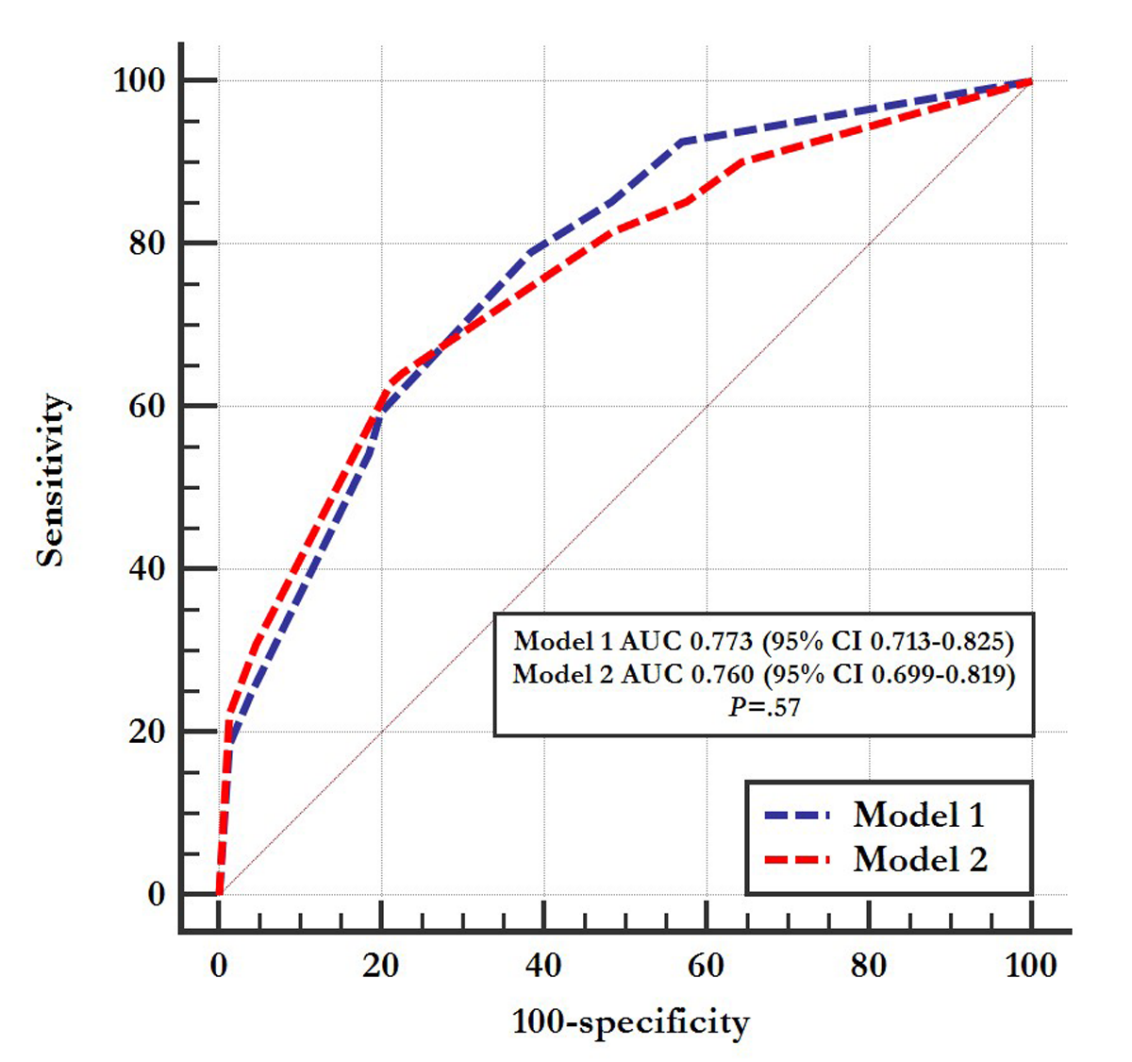
Receiver operating characteristic curves of the malnutrition, inflammation, and stroke model for 2-year survival prediction. AUC: area under the curve.

Table 4 shows the C statistics indicating the predictive performance level for 2-year longevity. The C statistic was 0.685 for the GNRI and 0.666 for the CONUT score initially. When CVA and NLR >3.89 were added to the regression model, it resulted in significant stepwise improvements of the C statistic from 0.685 to 0.773 in model 1 (*P*=.006) and 0.666 to 0.760 in model 2 (*P*=.004). However, adding CHF did not further improve the predictive value for 2-year longevity. The predictive performance levels for 2-year longevity was similar between the 2 models (0.773 vs 0.760; *P*=.57) (Figure 2).

**Table 4:** C statistics for the prediction of 2-year longevity.

Variable	Model 1		Model 2	
	C statistic (95% CI)	*P* value^a^	C statistic (95% CI)	*P* value^a^
GNRI^b^ <90.3	0.685 (0.621-0.744)	N/A^c^	N/A	N/A
CONUT^d^ score >3	N/A	N/A	0.666 (0.601-0.726)	N/A
Addition of CVA^a,e^	0.736 (0.675-0.792)	<.001	0.723 (0.660-0.779)	<.001
Further addition of NLR^a,f^ >3.89	0.773 (0.713-0.825)	.006	0.760 (0.699-0.813)	.004
Further addition of CHF^a,g^	0.774 (0.714-0.826)	.91	0.758 (0.698-0.812)	.86

^a^Compared with the previous model.

^b^GNRI: Geriatric Nutritional Risk Index.

^c^N/A: not applicable.

^d^CONUT: Controlling Nutritional Status.

^e^CVA: cerebrovascular accident.

^f^NLR: neutrophil-lymphocyte ratio.

^g^CHF: congestive heart failure.

**Figure 3:**
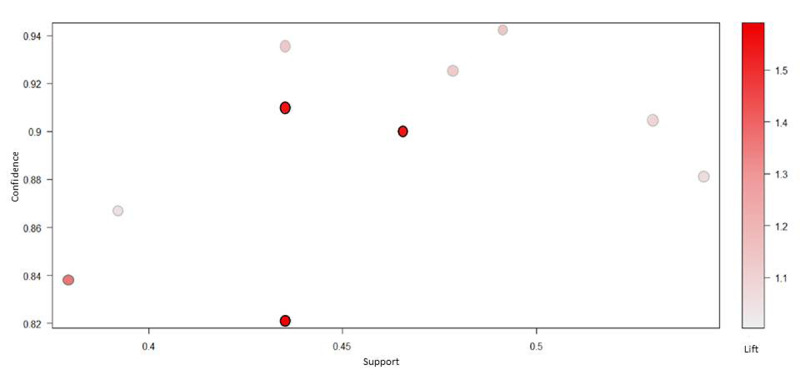
Top 10 association rules. The figure shows the plot with lift on the y-axis.

**Figure 4:**
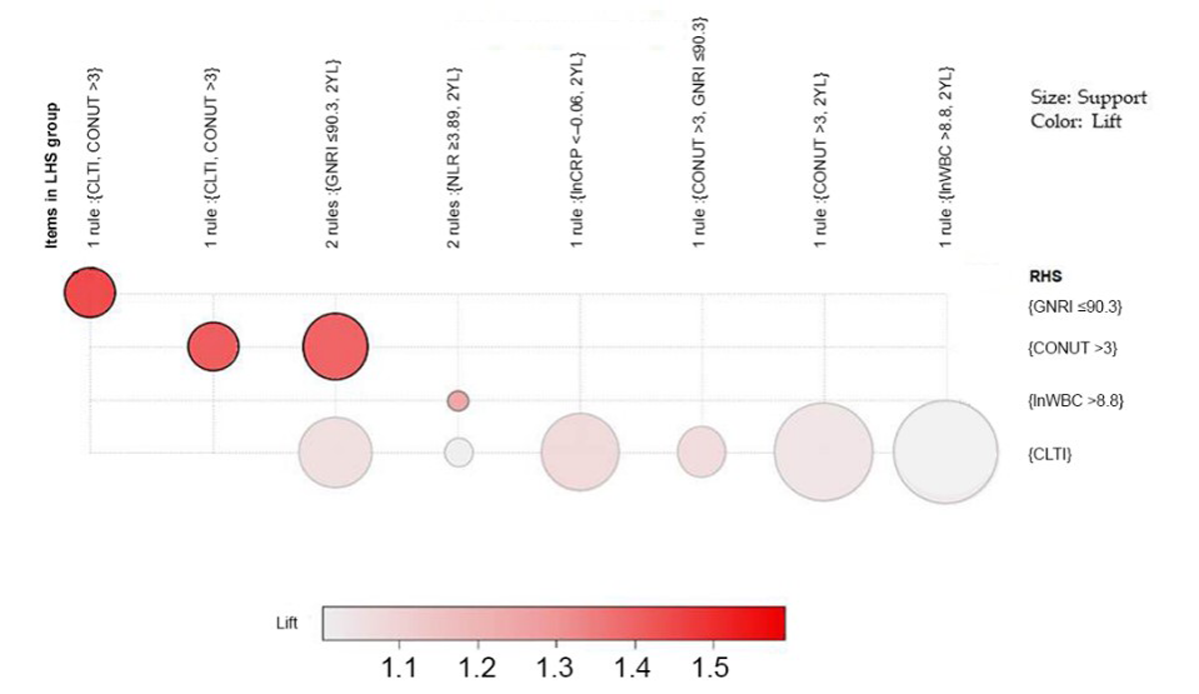
Grouped matrix for 10 rules. The graph-based visualization uses color or size to represent the set of items and rules. This graph offers a representation of rules and enables a small set of rules to avoid a cluttered presentation. The left-hand side shows the antecedents and the right-hand side shows the consequents. 2YL: 2-year longevity; CLTI: chronic limb-threatening ischemia; CONUT: Controlling Nutritional Status; CRP: C-reactive protein; GNRI: Geriatric Nutritional Risk Index; LHS: left-hand side; NLR: neutrophil-lymphocyte ratio; RHS: right-hand side; WBC: white blood cell.

We investigated 122 association rules based on 2-year longevity data. The top 10 association rules (Multimedia Appendix 4) are visually presented on a scatter plot (Figure 3). All rules with a high lift can be easily identified. Rules with a high lift typically have low support. The most interesting rules (support-confidence optimal rules) reside on the support-confidence border, which can be clearly seen in this plot. The association rules between 15 potential variables are ordered by support. Figure 4 shows the presented features on a grouped matrix of 10 associations. The following sets of items were interactively selected to reveal the preceding variables of rules and the following variable based on a grouped matrix for 10 rules: {GNRI, 2-year longevity} → {CONUT} and {NLR, 2-year longevity} → {lnWBC}. These data sets illustrated the close associations of malnutrition (GNRI, CONUT) and inflammation (NLR) factors with 2-year longevity in older patients.

**Figure 5:**
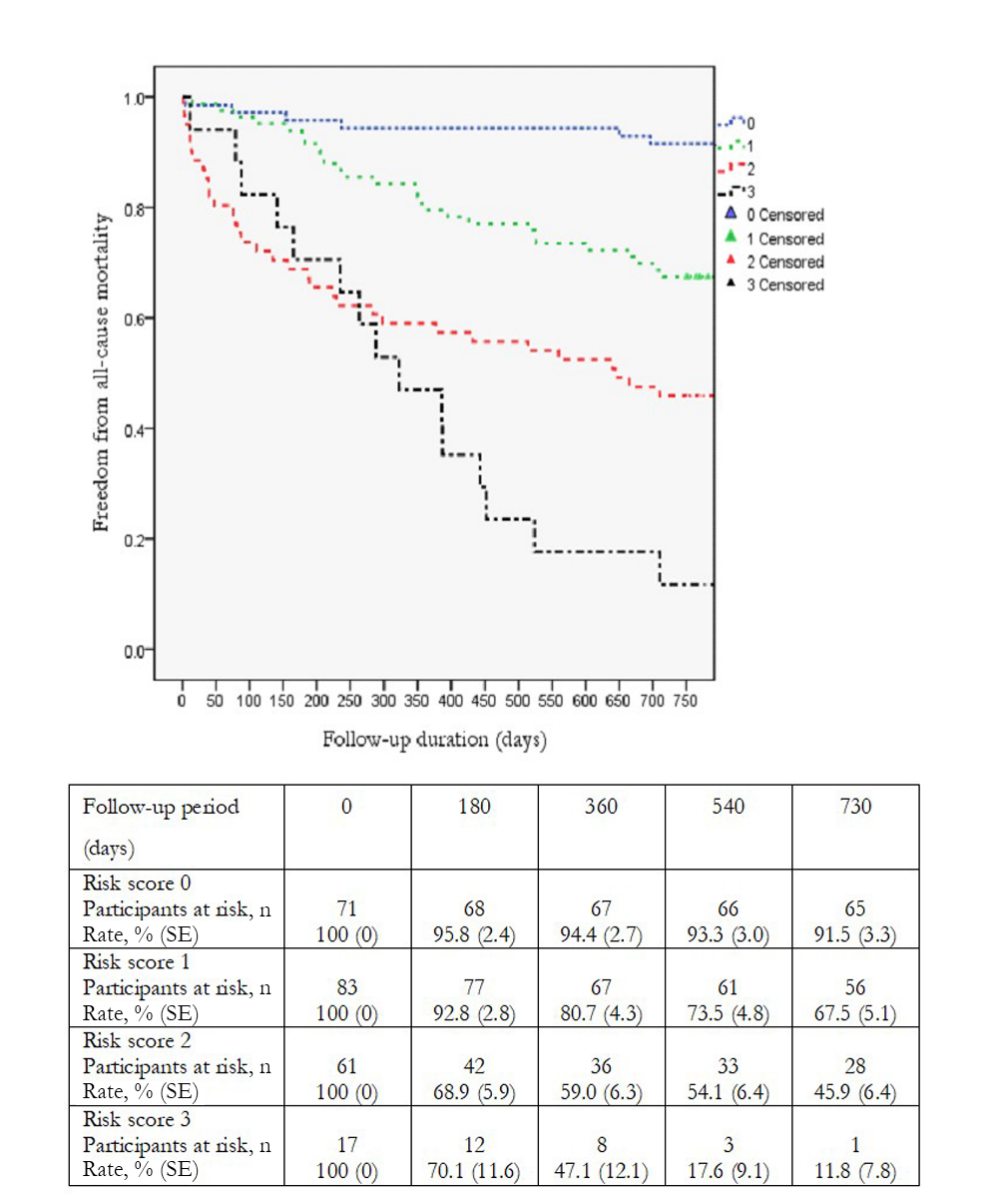
Kaplan-Meier analysis of 2-year survival in model 1.

**Figure 6:**
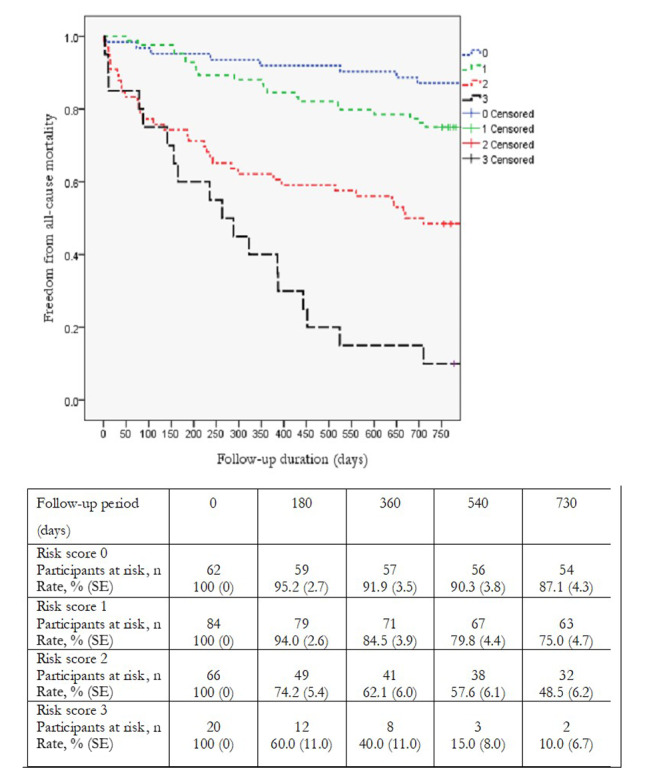
Kaplan-Meier analysis of 2-year survival in model 2.

Kaplan-Meier analysis demonstrated a significant decrease in 2-year survival as the number of malnutrition, inflammation, and stroke factors increased from 0 to 3 in both the GNRI-based model (92% vs 68% vs 46% vs 12%, respectively; *P*<.001) and the CONUT score model (87% vs 75% vs 49% vs 10%, respectively; *P*<0.001) (Figures 5 and 6), which was in line with the hazard ratio (HR) between patients with 3 factors and those without (HR 18.2, 95% CI 7.0-47.2; *P*<.001 in the GNRI model and HR 13.6, 95% CI 5.9-31.5; *P*<.001 in the CONUT model).

## Discussion

### Summary

This study demonstrated the association and predictive value of malnutrition, inflammation, and stroke factors in the 2-year survival of octogenarians or nonagenarians after treatment with EVT for LEAD. Simple prognostic stratification using malnutrition, inflammation, and stroke factors will help clinicians in deciding the appropriate treatment.

For symptomatic LEAD necessitating revascularization, 2-year longevity is a fundamental consideration in deciding the treatment option. Surgical bypass, recommended by the recent guidelines for patients surviving for more than 2 years [3,4], was underused in older patients [23,24] because of the high perioperative morbidity, complications, and poor long-term outcomes in these patients [5-7]. Although EVT was reported to be a safe procedure for older patients with LEAD [5,6,13] and older patients may benefit from endovascular revascularization, the factors affecting 2-year longevity remain uncertain.

### Association and Determining Variables for 2-Year Longevity

The ARA and grouped matrix method revealed that malnutrition and inflammation have meaningful roles in the 2-year longevity of octogenarians with atherosclerotic LEAD. The univariates affecting 2-year longevity were CHF, prior stroke, dialysis, ambulatory status, CLTI, BMI, total cholesterol, NLR, PLR, SII, CRP level, albumin, CONUT score, and GNRI. Those factors are clinically meaningful and consistent with prior reports by domain experts [25-28]. After adjusting for the covariates, NLR, prior stroke, and malnutrition (GNRI or CONUT score) remained independent predictors of 2-year longevity.

### Impact of Immune-Inflammation Factors on Survival

Several immune-inflammatory factors (NLR, PLR, and SII) have been reported as prognostic markers in patients with LEAD in various clinical settings [29-31]. Previous studies have reported that an NLR >5 increases 1- and 2-year mortality rates, and it has been used as a major component of the Valladolid Critical Limb Ischaemia Risk Scale (ERICVA) model to predict 1-year amputation-free survival after revascularization for CLTI [32-34]. In patients with claudication, the cut-off value for major adverse cardiac events and disease severity was around 3.05 to 3.3 [35,36]. Our cohort study of 232 patients incorporated 40 (17.2%) patients with claudication, and the cut-off value for NLR was 3.89.

Elevated NLR reflects both the neutrophilia of inﬂammation (mediated by arachidonic acid metabolites and platelet-aggregating factors, cytotoxic oxygen-derived free radicals, and hydrolytic enzymes) [37] and relative lymphopenia, suggesting a deeper imbalance in the immunologic response, an increased expression of T helper 17 over regulatory T cells, and the activation of the interleukin-17 axis, which in turn is associated with vascular dysfunction, the progression of atherosclerosis, and vascular events [38,39]. In our study, we found that NLR had a higher predictive performance level than PLR and SII. Although a previous report showed that NLR increases with age [40], a high NLR played an additional role in assessing 2-year longevity in older patients with LEAD.

### Nutrition

Impaired nutritional status, a functional disorder of frailty, increases the morbidity and mortality rates in older people [41]. Two objective indices of nutritional status, the GNRI and the CONUT score, have been reported as prognostic factors in patients with CLTI after EVT treatment [17,42]. It was found that patients with moderate to severe malnutrition (GNRI of <91-92 or CONUT of ≥4) had significantly higher cardiovascular and limb events compared with patients with normal nutritional status. The GNRI cut-off value of 90.3 was slightly lower in our study, which reflects a lower BMI in older patients with malnutrition.

Chronic limb ischemia increases cytokine release, oxidative stress, and inflammatory cell accumulation, leading to malnutrition by affecting appetite, wasting resting energy, and increasing muscle protein breakdown [43,44]. Furthermore, malnutrition aggravates the progression of atherosclerosis. This cycle is called malnutrition-inflammation-atherosclerosis syndrome [44]. In this study, C statistics further validated the prognostic implication of these factors for 2-year survival, indicating the crucial role of malnutrition and impaired immune defenses in older people with preexisting atherosclerotic disease.

### Comparisons With Prior Work

Moxey et al [25] compared the BASIL survival prediction model to the Finland National Vascular registry and Prevention of Infrainguinal Vein Graft Failure III (PREVENT III) models. The power of each model to predict mortality was evaluated by comparing the AUC for each ROC curve. The AUCs for 2-year mortality ranged from 0.533 to 0.664 in those models, which indicates weak to good prediction. In our study, the combination of independent covariates using C statistics further increased the AUC and determined the predictive value for 2-year longevity estimation.

**Figure 7:**
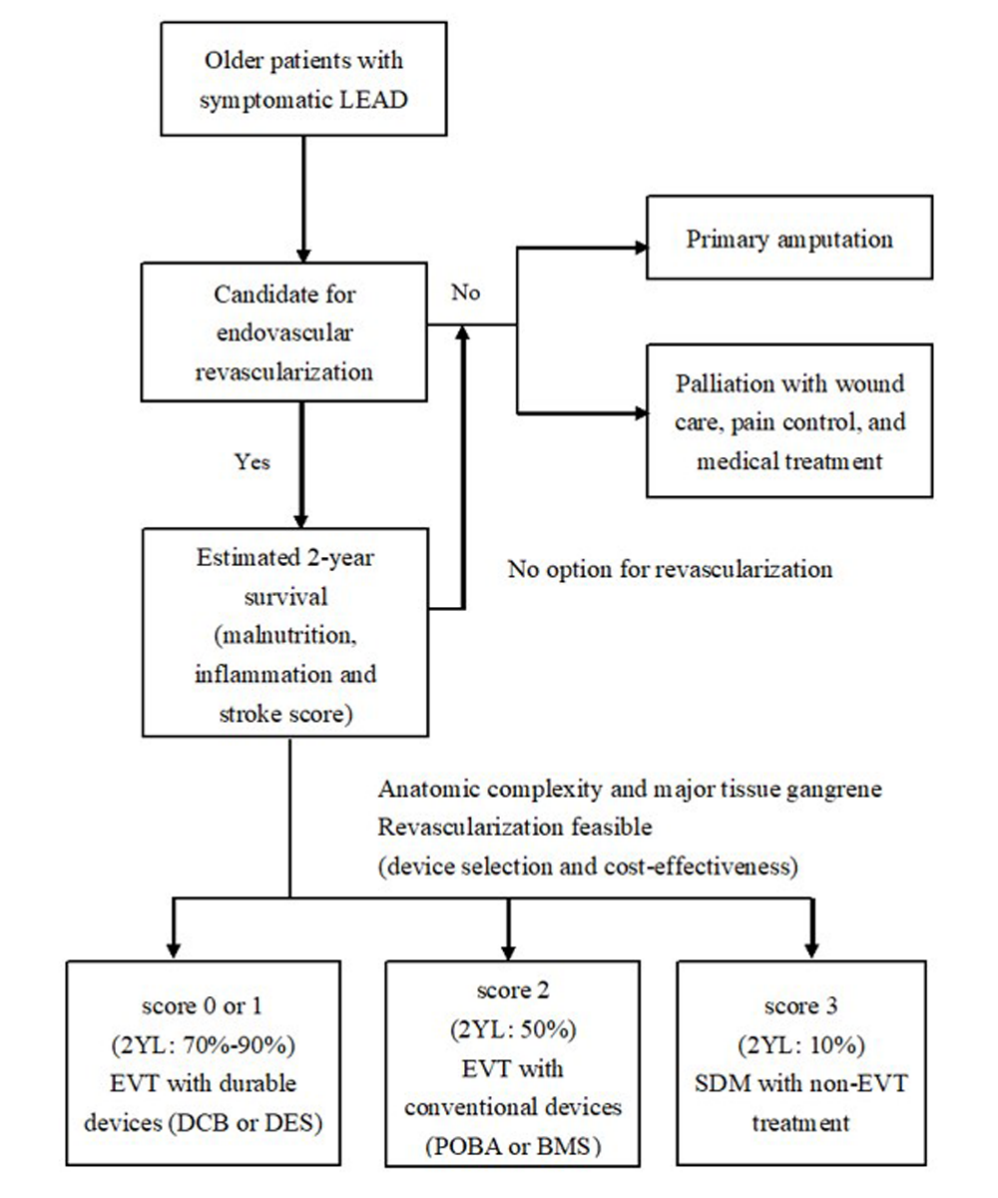
Treatment framework using the malnutrition, inflammation, and stroke score for older patients with symptomatic lower extremity arterial disease. 2YL: 2-year longevity; BMS: bare-metal stent; DCB: drug-coated balloon; DES: drug-eluting stent; EVT: endovascular therapy; LEAD: lower extremity arterial disease; POBA: plain old balloon angioplasty; SDM: shared decision making.

Effective revascularization is the cornerstone treatment for patients with symptomatic LEAD, and EVT is the first-line treatment strategy in older people due to lower procedural risk. To aid the clinical decision making in daily practice, we used the malnutrition, inflammation, and stroke scores for risk stratification. Anatomical complexity and wound status, which are limb outcome predictors, help health care professionals select appropriate devices to treat limb ischemia and optimize the EVT results. Figure 7 illustrates the framework of the treatment decision. For patients without any or with only 1 malnutrition, inflammation, and stroke factor, 2-year longevity is around 70% to 90%. Thus, EVT should be performed and durable drug-coated devices should be applied to treat limb ischemia because of the longer life expectancy. For patients with 2 malnutrition, inflammation, and stroke factors, 2-year longevity is around 50%. EVT using standard balloon angioplasty or bare-metal stents might be cost-effective, as long-term vascular patency is not the focus for patients with an average life expectancy.

In contrast, 2-year longevity is only 10% in patients with 3 malnutrition, inflammation, and stroke factors. These patients may be appropriately treated with primary amputation or non-EVT treatment, such as spinal cord stimulation, lumbar sympathectomy, or intermittent pneumatic compression. Health care professionals can share their decision making with patients, families, and caregivers, who should have access to appropriate expertise when dealing with these challenging scenarios. This approach balances life expectancy, invasiveness, and benefits, which is consistent with the PLAN (patient risk estimation, limb staging, and anatomic pattern of arterial disease) concept recommended by current practical guidelines [45].

### Study Limitations

This study had several limitations. First, this was an observational cohort study using a prospective database and all patients were treated in a single institution, indicating a potential selection bias. Second, the patient enrollment period spanned more than 10 years, and the outcomes and risk models may have changed over time due to new techniques, management, or reimbursement policies. Third, this study lacks data on subsequent changes in nutritional status. Therefore, we cannot determine whether nutrition status change had any effect on the clinical outcome of patients with peripheral arterial disease. Fourth, patients with poor cognitive function and uncooperative patients with dementia were not enrolled, which might have affected the results. We also did not enroll patients undergoing surgical revascularization, and outcome differences could not be determined between EVT and bypass surgery using the same scoring techniques. Despite the useful information regarding malnutrition, inflammation, and stoke scores in clinical practice, global assessment and shared decision making plays a crucial role in judging the balance between risk and effectiveness when dealing with challenging scenarios. Large-scale trials to prove the benefit of malnutrition, information, and stroke scores are warranted. Finally, ARA data mining techniques cannot rank association rules or determine their weight, and no measures of the importance of items in a rule or rule set have been proposed thus far [46].

### Conclusions

Malnutrition, inflammation, and stroke factors are associated with 2-year longevity and play a crucial role in assessing older patients with LEAD. The presence of each malnutrition, inflammation, and stroke factor or their combination worsens the survival potential of these patients. Awareness of the likelihood of intermediate-term survival better informs the discussion with patients about the beneﬁts of EVT, its attendant risks, and intermediate-term outcomes.

## Appendix

Multimedia Appendix 1 Receiver-operating-characteristic curves of inflammatory variables for 2-year survival prediction.

Multimedia Appendix 2 Supplementary Table 1. Patient demographics.

Multimedia Appendix 3 Supplementary Table 2. Lesion and interventional procedure characteristics.

Multimedia Appendix 4 Top 10 association rules of clinical data.

## Abbreviations

ARA: association rules analysis
AUC: area under the curve
BASIL: Bypass Versus Angioplasty in Severe Ischaemia of the Leg
CBC: complete blood count
CHF: congestive heart failure
CLTI: chronic limb-threatening ischemia
CONUT: Controlling Nutritional Status
CRP: C-reactive protein
CVA: cerebrovascular accident
ERICVA: Valladolid Critical Limb Ischaemia Risk Scale
EVT: endovascular therapy
GNRI: Geriatric Nutritional Risk Index
HR: hazard ratio
LEAD: lower extremity arterial disease
NLR: neutrophil-lymphocyte ratio
PLAN: patient risk estimation, limb staging, and anatomic pattern of arterial disease
PLR: platelet-lymphocyte ratio
PREVENT III: Prevention of Infrainguinal Vein Graft Failure III
ROC: receiver operating characteristic
SII: systemic immune-inflammation index
WBC: white blood cell
