# Supplementary figures and images for: Association Rule Mining and Prognostic Stratification of 2-Year Longevity in Octogenarians Undergoing Endovascular Therapy for Lower Extremity Arterial Disease: Observational Cohort Study
Source: J Med Internet Res. 2020 Dec 1;22(12):e17487. doi: 10.2196/17487 (PMC7909897; doi:10.2196/17487)

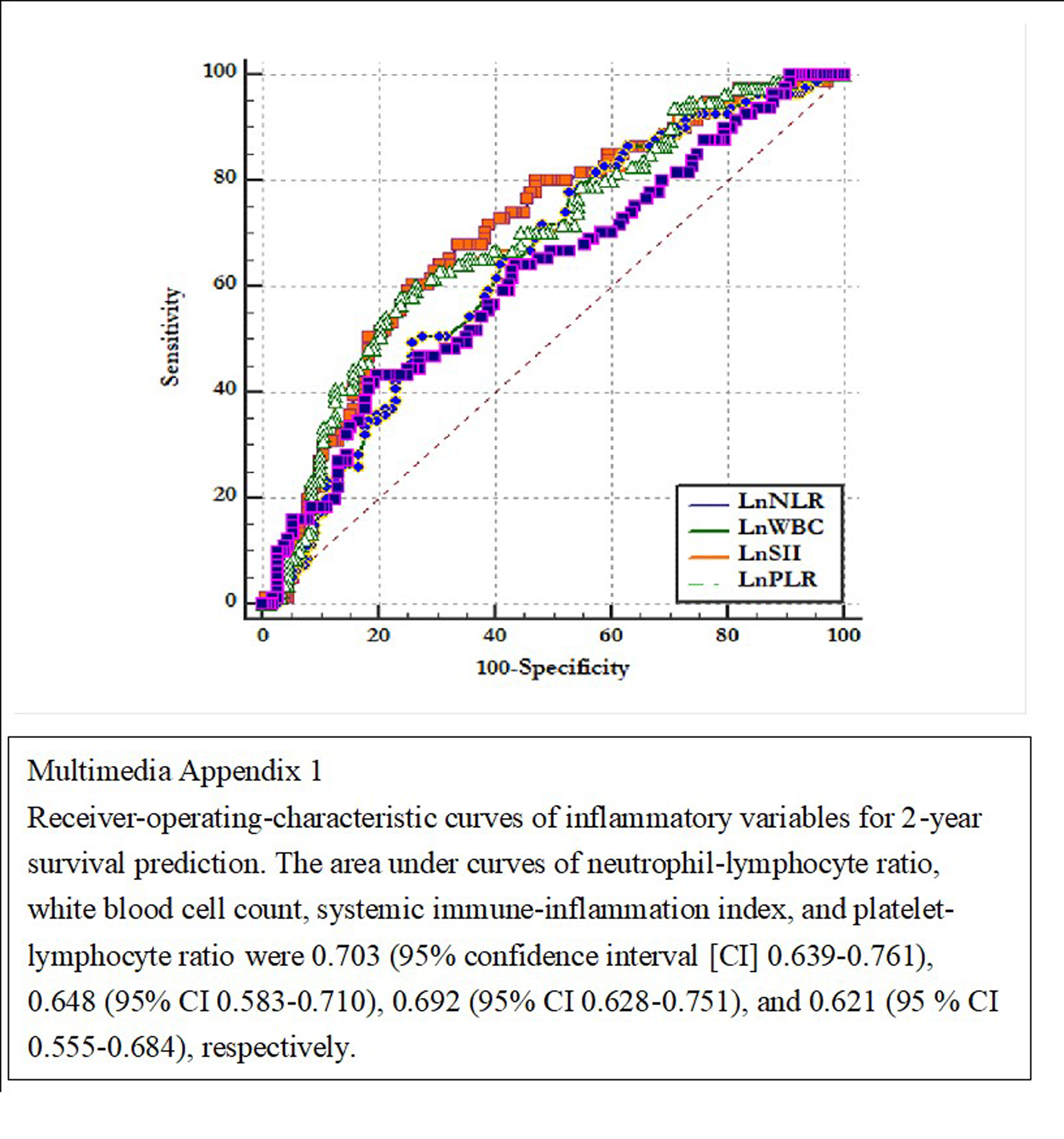

Supplement: Multimedia Appendix 1 [file jmir_v22i12e17487_app1.png]
