# Supplementary material for: Association Rule Mining and Prognostic Stratification of 2-Year Longevity in Octogenarians Undergoing Endovascular Therapy for Lower Extremity Arterial Disease: Observational Cohort Study
Source: J Med Internet Res. 2020 Dec 1;22(12):e17487. doi: 10.2196/17487 (PMC7909897; doi:10.2196/17487)
Supplement: Multimedia Appendix 2 [file jmir_v22i12e17487_app2.pdf]

Supplementary Table 1. Patient demographics

Factors	All patients	Group A (2-year death)	Group B (2-year survival)	*P*-Value
Number of patients	232	81	151	
Age (years)	85.4±4.2	85.8±4.0	85.3±4.3	0.408
Sex (male)	109 (47%)	36 (44%)	73 (48%)	0.570
Diabetes mellitus	130 (56%)	51 (62%)	79 (52%)	0.119
Hypertension	201 (87%)	72 (89%)	129 (85%)	0.460
Coronary artery disease	93 (40%)	32 (40%)	61 (40%)	0.895
Congestive heart failure	39 (17%)	19 (24%)	20 (13%)	0.047
Cerebrovascular accident	52 (22%)	29 (36%)	22 (15%)	<0.001
Chronic kidney disease	160 (69%)	58 (72%)	102 (68%)	0.524
Dialysis dependence	57 (25%)	27 (33%)	30 (20%)	0.023
Smoking history	58 (25%)	19 (24%)	39 (26%)	0.691
Atrial fibrillation	49 (21%)	20 (25%)	29 (19%)	0.329
Hyperlipidemia	95 (41%)	31 (39%)	64 (42%)	0.593
Ambulatory status	83 (36%)	16 (20%)	67 (44%)	<0.001
Critical threatening-limb ischemia	192 (83%)	78 (96%)	114 (76%)	<0.001
Tissue loss	141 (61%)	59 (73%)	82 (54%)	0.006
Body mass index (kg/m^2)	23.1±3.47	22.3±3.48	23.6±3.39	0.006
Cholesterol (mg/dL)	159±39	150±39	163±39	0.014
Triglyceride (mg/dL)	117±70	111±63	119±73	0.459

High-density lipoprotein cholesterol (mg/dL)	43±14	41±14	44±15	0.144
Low-density lipoprotein cholesterol (mg/dL)	94±33	89±33	96±32	0.147
Glycohemoglobin (%)	6.51±1.50	6.70±1.68	6.42±1.41	0.211
Hematocrit[a] (%)	33.7 (29.8, 37.2)	33.5 (29.9, 36.5)	33.9 (29.8, 38.1)	0.095
White blood cell count[a] (10^9/L)	7.230 (5.673, 9.080)	7.970 (6.720, 9.965)	6.850 (5.120, 8.200)	0.001
Neutrophil count[a] (10^9/L)	4.896 (3.602, 6.921)	5.880 (4.324, 7.963)	4.324 (3.127, 5.882)	<0.001
Lymphocyte count[a] (10^9/L)	1.362 (1.025, 1.722)	1.260 (0.999, 1.284)	1.446 (1.062, 1.809)	0.043
Platelet count[a] (10^3/μL)	208 (164, 255)	217 (169, 278)	205 (156, 245)	0.066
Neutrophil:lymphocyte ratio[a]	3.62 (2.34, 5.62)	5.03 (3.31, 7.02)	3.03 (2.04, 4.64)	<0.001
Platelet:lymphocyte ratio[a]	145 (114, 219)	173 (120, 243)	135 (107, 195)	0.002
Systemic immune-inflammation index[a]	716 (461, 1272)	1083 (568, 1835)	598 (383, 972)	<0.001
C-reactive protein[a] (mg/dL)	1.25 (0.31, 4.40)	3.08 (0.77, 8.20)	0.90 (0.19, 2.92)	<0.001
Albumin (g/dL)	3.10±0.65	2.78±0.63	3.26±0.59	<0.001
CONUT score	4.74±2.97	6.26±2.81	3.93±2.72	<0.001
Geriatric Nutritional Risk Index	89.3±12.5	83.1±12.3	92.6±11.3	<0.001
Medication				
Aspirin	139 (64%)	43 (63%)	96 (64%)	0.961
Clopidogrel	156 (71%)	51 (75%)	105 (70%)	0.409
Cilostazol	165 (75%)	48 (71%)	117 (78%)	0.273
ACEI or ARB	98 (45%)	28 (42%)	70 (46%)	0.532
Statin	44 (20%)	13 (20%)	31 (21%)	0.809
Beta-blocker	83 (38%)	28 (41%)	55 (36%)	0.502

Calcium channel blocker	96 (44%)	26 (38%)	70 (46%)	0.262
Insulin	36 (16%)	14 (21%)	22 (15%)	0.266
Median follow-up time (days)	971 (389, 1575)	229 (79, 438)	1360 (1008, 1942)	<0.001
Causes of death	138	81	57	
Cardiac	43 (31%)	27 (33%)	16 (28%)	0.511
Non-cardiac	95 (69%)	54 (67%)	41 (72%)	

Values are mean±standard deviation or n (%).

[a]Expressed as median and interquartile range.

CKD was defined as estimated glomerular filtration rate <60 mL/min/1.73 m^2 with dialysis dependence.

Systemic immune-inflammation index was defined as platelet count × neutrophil count/lymphocyte count.

Abbreviations: ACEI, angiotensin-converting enzyme; ARB, angiotensin receptor blocker; CONUT, Controlling Nutritional Status
