# Supplementary material for: Association Rule Mining and Prognostic Stratification of 2-Year Longevity in Octogenarians Undergoing Endovascular Therapy for Lower Extremity Arterial Disease: Observational Cohort Study
Source: J Med Internet Res. 2020 Dec 1;22(12):e17487. doi: 10.2196/17487 (PMC7909897; doi:10.2196/17487)
Supplement: Multimedia Appendix 3 [file jmir_v22i12e17487_app3.pdf]

Supplementary Table 2. Lesion and interventional procedure characteristics

Factors	All limbs	Group A (2-year death)	Group B (2-year survival)	*P*-Value
Limb number	N=232	n=81	n=151	
Claudication	40 (17%)	3 (4%)	37 (25%)	0.001
Resting pain	51 (22%)	19 (23%)	32 (21%)	
Unhealing ulcer	109 (47%)	44 (54%)	65 (43%)	
Gangrene	32 (14%)	15 (19%)	17 (11%)	
ABI of affected limbs	0.54±0.33	0.51±0.37	0.55±0.31	0.403
ABI of affected limbs excluding ≥1.4	0.45±0.19	0.41±0.18	0.47±0.19	0.016
TASC II classification				
Iliac lesions	30 (12.9%)	9 (11.1%)	21 (13.9%)	0.980
A	7 (3.0%)	2 (2.5%)	5 (3.0%)	
B	7 (3.0%)	2 (2.5%)	5 (3.0%)	
C	7 (3.0%)	2 (2.5%)	5 (3.0%)	
D	9 (3.9%)	3 (3.7%)	6 (3.9%)	
Femoropopliteal lesions	167 (72.0%)	61 (75.3%)	106 (70.2%)	0.353

A	7 (2.8%)	1 (1.2%)	6 (4.0%)	
B	63 (32.8%)	24 (29.6%)	39 (25.8%)	
C	48 (19.9%)	20 (24.7%)	28 (18.5%)	
D	42 (10.4%)	13 (16.0%)	29 (19.2%)	
In-stent restenosis	2 (0.7%)	0	2 (1.3%)	
In-stent occlusion	5 (0.6%)	3 (3.7%)	2 (1.3%)	
Below-the-knee lesions	170 (73.3%)	61 (75.3%)	109 (73.2%)	0.630
A	14 (6.0%)	6 (7.4%)	8 (5.3%)	
B	15 (6.5%)	4 (4.9%)	11 (7.3%)	
C	36 (15.5%)	16 (19.8%)	20 (13.2%)	
D	105 (45.3%)	35 (43.2%)	70 (46.4%)	
Isolated EVT				
Iliac	14 (6.0%)	3 (3.7%)	11 (7.3%)	0.275
Femoropopliteal	38 (16.4%)	14 (17.3%)	24 (15.9%)	0.785
Below the knee	51 (22.0%)	17 (21.0%)	34 (22.5%)	0.789
Multi-level EVT				

Iliac+FP	10 (4.3%)	3 (3.8%)	7 (4.6%)	0.753
Iliac+FP+BTK	6 (2.6%)	3 (3.8%)	3 (2.0%)	0.433
Iliac+BTK	0	0	0	
FP+BTK	113 (49.3%)	41 (51.3%)	72 (48.3%)	0.673
Poor runoff	192 (83.8%)	67 (84.8%)	125 (83.3%)	0.773
Stenting	112 (48.3%)	37 (45.7%)	75 (49.7%)	0.526
Lesion score	3.18±0.98	3.30±0.97	3.11±0.98	0.164
Number of pedal arch vessels before EVT	1.08±0.60	1.08±0.58	1.08±0.61	0.971
Number of pedal arch vessels after EVT	1.42±0.50	1.44±0.50	1.41±0.51	0.749

Values are mean±standard deviation or n (%).

Abbreviations: ABI, ankle-brachial index; BTK, below the knee; EVT, endovascular therapy; FP, femoropopliteal; TASC, Trans-Atlantic Inter-society Consensus
