# Supplementary material for: Association Rule Mining and Prognostic Stratification of 2-Year Longevity in Octogenarians Undergoing Endovascular Therapy for Lower Extremity Arterial Disease: Observational Cohort Study
Source: J Med Internet Res. 2020 Dec 1;22(12):e17487. doi: 10.2196/17487 (PMC7909897; doi:10.2196/17487)
Supplement: Multimedia Appendix 4 [file jmir_v22i12e17487_app4.docx]

Supplementary Table 3: Top 10 association rules of clinical data

| No | Association rules | Support | Confidence |
| --- | --- | --- | --- |
| 1 | Conut>3 => CLTI | 0.5301724 | 0.9044118 |
| 2 | CLTI => Conut>3 | 0.5301724 | 0.6406250 |
| 3 | 2YL => CLTI | 0.4913793 | 0.7549669 |
| 4 | CLTI => 2YL | 0.4913793 | 0.5937500 |
| 5 | LnCRP>-0.06 => CLTI | 0.4913793 | 0.9421488 |
| 6 | CLTI => LnCRP>-0.06 | 0.4913793 | 0.5937500 |
| 7 | GNRI≤90.3=> CLTI | 0.4784483 | 0.9250000 |
| 8 | CLTI => GNRI≤90.3 | 0.4784483 | 0.5781250 |
| 9 | GNRI≤90.3=> Conut>3 | 0.4655172 | 0.9000000 |
| 10 | Conut>3=> GNRI≤90.3 | 0.4655172 | 0.7941176 |

Abbreviation: CLTI, chronic limb-threatening ischemia; CONUT, controlling nutritional status; CRP, C-reactive protein; GNRI, geriatric nutritional risk index;

2YL, two-year longevity
